# Machado-Joseph disease in a Sudanese family links East Africa to Portuguese families and allows reestimation of ancestral age of the Machado lineage

**DOI:** 10.1007/s00439-023-02611-8

**Published:** 2023-11-14

**Authors:** Sandra Martins, Ashraf Yahia, Inês P. D. Costa, Hassab E. Siddig, Rayan Abubaker, Mahmoud Koko, Marc Corral-Juan, Antoni Matilla-Dueñas, Alexis Brice, Alexandra Durr, Eric Leguern, Laura P. W. Ranum, António Amorim, Liena E. O. Elsayed, Giovanni Stevanin, Jorge Sequeiros

**Affiliations:** 1 i3S—Instituto de Investigação e Inovação em Saúde, University of Porto, Porto, Portugal; 2 IPATIMUP—Institute of Molecular Pathology and Immunology, University of Porto, Porto, Portugal; 3 Faculty of Medicine, University of Khartoum, Khartoum, Sudan; 4 Sorbonne University, Paris Brain Institute - ICM, Inserm, CNRS, AP-HP, Paris, France; 5 Center of Neurodevelopmental Disorders (KIND), Centre for Psychiatry Research, Department of Women’s and Children’s Health, Karolinska Institutet and Stockholm Health Care Services, Stockholm, Sweden; 6 Division of Neurology, Sudan Medical Council, Khartoum, Sudan; 7 Sudanese Neurogenetics Research Group, Faculty of Medicine, University of Khartoum, Khartoum, Sudan; 8 Functional and Translational Neurogenetics Unit, Department of Neuroscience, Health Sciences Research Institute Germans Trias I Pujol (IGTP)-Universitat Autònoma de Barcelona, Can Ruti Campus, Badalona, Barcelona, Spain; 9 Genetics Department, APHP, Pitié-Salpêtrière Hospital, Paris, France; 10 Center for NeuroGenetics, College of Medicine, University of Florida, Gainesville, FL USA; 11 Department of Basic Sciences, College of Medicine, Princess Nourah bint Abdulrahman University, Riyadh, Saudi Arabia; 12 Univ. Bordeaux, CNRS—Centre National de la Recherche Scientifique, INCIA—Institut de Neurosciences Cognitives et Intégratives d’Aquitaine, UMR 5287, Bordeaux, France; 13 EPHE—École Pratique des Hautes Études, CNRS—Centre National de la Recherche Scientifique, INCIA—Institut de Neurosciences Cognitives et Intégratives d’Aquitaine, UMR 5287, Paris, France; 14 UnIGENe and CGPP—Centro de Genética Preditiva e Preventiva, IBMC—Institute for Molecular and Cell Biology, University of Porto, Porto, Portugal; 15 ICBAS School of Medicine and Biomedical Sciences, University of Porto, Porto, Portugal; 16 Department of Biology, Faculty of Sciences, University of Porto, Porto, Portugal

## Abstract

Machado-Joseph disease (MJD/SCA3) is the most frequent dominant ataxia worldwide. It is caused by a (CAG)_n_ expansion. MJD has two major ancestral backgrounds: the Machado lineage, found mainly in Portuguese families; and the Joseph lineage, present in all five continents, probably originating in Asia. MJD has been described in a few African and African-American families, but here we report the first diagnosed in Sudan to our knowledge. The proband presented with gait ataxia at age 24; followed by muscle cramps and spasticity, and dysarthria, by age 26; he was wheel-chair bound at 29 years of age. His brother had gait problems from age 20 years and, by age 21, lost the ability to run, showed dysarthria and muscle cramps. To assess the mutational origin of this family, we genotyped 30 SNPs and 7 STRs flanking the *ATXN3*_CAG repeat in three siblings and the non-transmitting father. We compared the MJD haplotype segregating in the family with our cohort of MJD families from diverse populations. Unlike all other known families of African origin, the Machado lineage was observed in Sudan, being shared with 86 Portuguese, 2 Spanish and 2 North-American families. The STR-based haplotype of Sudanese patients, however, was distinct, being four steps (2 STR mutations and 2 recombinations) away from the founder haplotype shared by 47 families, all of Portuguese extraction. Based on the phylogenetic network constructed with all MJD families of the Machado lineage, we estimated a common ancestry at 3211 ± 693 years ago.

## Introduction

Machado-Joseph disease (MJD) is a late-onset neurological disease, characterized mainly by gait ataxia, usually followed by loss of coordination in lower limbs, dysarthria, dystonia, pseudobulbar or bulbar dysphonia, and progressive external ophthalmoplegia (Coutinho and Andrade 1978; Lima and Coutinho 1980). The high clinical pleomorphism of the disease led Coutinho and Andrade (1978) to propose three main subphenotypes in MJD: type 2, the most common, with progressive cerebellar ataxia, pyramidal signs and external ophthalmoplegia, with mean age-at-onset (AO) at 40.5 years; type 1, more severe, with additional extrapyramidal and marked pyramidal signs (mean AO, 24.3 years); and type 3, with additional prominent distal muscular atrophies and sensory loss and slower progression (mean AO, 46.8 years) (Coutinho 1992; Coutinho and Andrade 1978).

MJD belongs to the clinically and genetically heterogeneous group of autosomal dominant spinocerebellar ataxias (SCA), and is known also as SCA3. The causative gene, *ATXN3* (14q32.12), contains a highly polymorphic CAG repeat tract, expanded above 61 units in patients (Kawaguchi et al. 1994; Maciel et al. 2001). Although first described among descendants of Portuguese immigrants in the United States, MJD is currently known in many populations, with variable relative frequency among the SCAs, but being (overall) the most frequent dominant ataxia worldwide (Sequeiros et al. 2012).

In Africa, MJD has been reported from Morocco, Algeria, Mali, Ivory Coast, Ghana, Nigeria and Somalia, as well as in several African-American patients (Buhmann et al. 2003; Gaspar et al. 2001; Healton et al. 1980; Martins et al. 2007, 2012; Ogun et al. 2015; Subramony et al. 2002; Traore et al. 2011). Interestingly, a Parkinsonian phenotype (named MJD type 4), rarely observed in European patients, was shown to be common in families of African descent, including the first African-American family in which MJD was suspected (Gwinn-Hardy et al. 2001; Healton et al. 1980; Rosenberg 1983; Subramony et al. 2002). This fact led to the hypothesis of a common origin shared by MJD families of African descent; however, a common haplotypic background was not observed (Ogun et al. 2015; Subramony et al. 2002).

Previously, we had studied the ancestral origins of MJD in almost 300 families from over 20 populations (Gaspar et al. 2001; Li et al. 2018; Martins et al. 2007, 2012; Martins and Sequeiros 2018; Ogun et al. 2015; Sharony et al. 2019), and suggested that two main de novo expansions must have occurred at *ATXN3*, followed by different routes of migration, responsible for the MJD distribution observed worldwide. (1) The Joseph lineage, predominant in the Portuguese-Azorean island of Flores (birthplace of the Joseph family), including a few phylogenetically close “Joseph-like” sublineages, seem to share a common mutational event, which probably occurred in Asia, 6,000 to 16,000 years ago (Li et al. 2018; Martins et al. 2007, 2012). (2) The Machado lineage and some “Machado-like” sublineages are associated with MJD families from the Portuguese-Azorean island of São Miguel (birthplace of the Machado kindred), and seem to have a much more restricted geographic distribution, with an estimated age of 1,416 ± 434 years (based solely on 4 STRs flanking the CAG repeat) (Martins et al. 2007).

Here, we report a family with MJD, to our knowledge the first diagnosed in Sudan, as the only African MJD family to share the Machado lineage described up to now. After assessing intragenic SNP and flanking STR haplotypes, we were also able to estimate more accurately the age of the Machado lineage mutational event.

## Subjects and methods

**Fig. 1:**
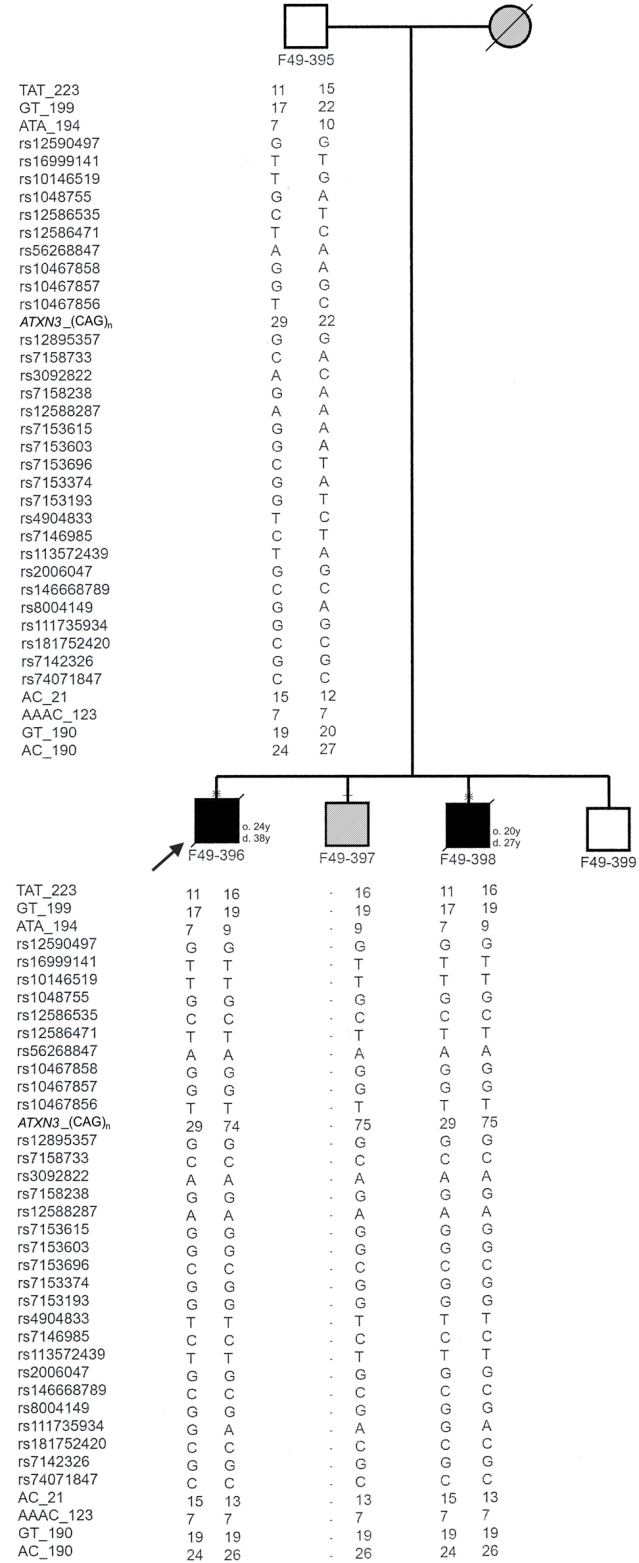
Pedigree of a Sudanese family affected with Machado-Joseph disease, showing the haplotypes of 30 SNPs and 7 STRs segregating with *ATXN3*_(CAG)_n_ alleles in 3 siblings and their unaffected father. The proband is marked with an arrow. Individual F49-397 had several inconsistencies in his paternal haplotype (not shown), which were not possible to clarify further. Ages of onset (o.) and death (d.) are described next to the symbol of the respective patient. Individuals sampled for haplotype analysis are marked with a dash above the symbol; in case a neurological exam was performed, an X sign was added. Patients affected by history have hatched symbols

**Fig. 2:**
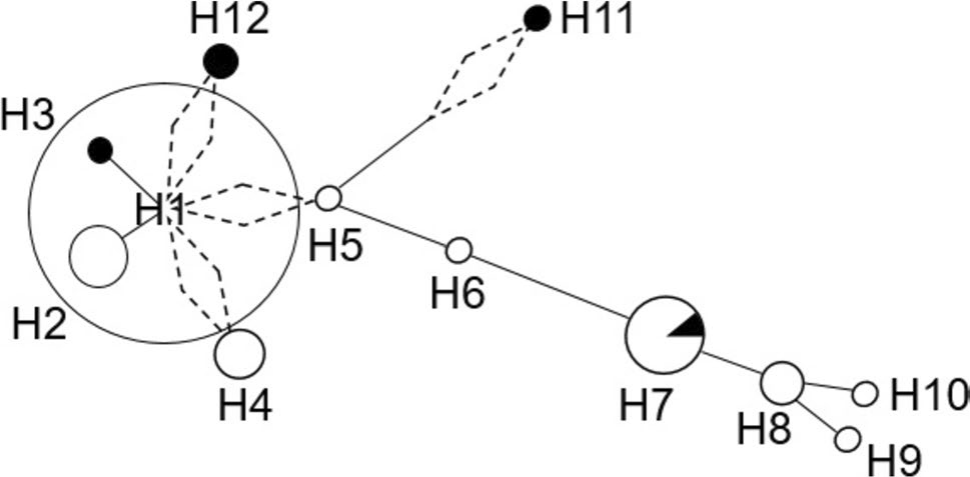
Phylogenetic network showing the most parsimonious relationships among haplotypes of 7 STRs in 82 MJD families of the Machado lineage. Circle size is proportional to number of families. The length of lines reflect the number of stepwise mutations. Dashed diamonds indicate recombination. Non-Portuguese families are represented in black: 2 from the USA (H3 and H7), 2 Spanish (H12); and 1 Sudanese (H11)

Two siblings from a Sudanese family (F49-396 and F49-398) were examined neurologically by Drs. Ashraf Yahia, Hassab Elrasoul Siddig and Mahmoud Koko. This family was part of a cohort of Sudanese families with hereditary spinocerebellar degenerations (Yahia et al. 2023). At the time of examination, in 2015, patients were 33 and 24 years old, respectively. Their two other sibs F49-397 and F49-399, aged 30 and 19 years at examination time, were asymptomatic and had a normal neurological exam. At the present time, individual F49-397 is known to have developed symptoms of MJD, although no clinical evaluation has been performed. The deceased mother (affected by history) had no health records or genetic test for *ATXN3*_(CAG)_n_. Informed written consents were obtained from analysed individuals. We genotyped 3 siblings (F49-396–398) and their non-affected father (F49-395) (Fig. 1) to assess 30 intragenic SNPs (26 within a 4 kb region flanking the (CAG)_n_ and 4 about 12 kb upstream), for the identification of MJD lineages (Table 1). We also typed 7 flanking STRs (4 di-, 2 tri-, and 1 tetranucleotidic), in a region of ~ 414 kb (223 up and 191 downstream the CAG repeat) to construct the phylogenetic tree and estimate the age of the mutational event (Table 2, Fig. 2), from the diversity accumulated since the ancestral haplotype H1 (Costa et al. 2019; Martins et al. 2007). The PHASEv2.1.1 software (Stephens et al. 2001) was used to infer haplotypes (threshold probability > 0.6) from genotypic data when the complete allelic phase was not directly assessed by segregation.

**Table 1:** SNP-based haplotypes (15 kb) of the Sudanese family with MJD, compared with the main Joseph and Machado MJD mutational origins (refSNPs according to Genome Reference Consortium GRCh38.p14)

dbSNP ID	refSNP (HGVS)	Joseph lineage	Machado lineage	Sudanese family	
rs12590497	NC_000014.9:g.92083288 C > A	T	G	G	
rs16999141	NC_000014.9:g.92083242 G > A	T	T	T	
rs10146519	NC_000014.9:g.92082552 A > C	G	T	T	
rs1048755	NC_000014.9:g.92082441 C > T	A	G	G	
rs12586535	NC_000014.9:g.92072185 G > A	T	C	C	
rs12586471	NC_000014.9:g.92072061 A > G	C	T	T	
rs56268847	NC_000014.9:g.92071824 T > C	A	A	A	
rs10467858	NC_000014.9:g.92071441 C > T	A	G	G	
rs10467857	NC_000014.9:g.92071399 G > C	G	G	G	
rs10467856	NC_000014.9:g.92071388 A > G	C	T	T	
	(CAG)_n_				
rs12895357	NC_000014.9:g.92071010 C > G	C	G	G	
rs7158733	NC_000014.9:g.92070879 G > T	A	C	C	
rs3092822	NC_000014.9:g.92070819 T > G	C	A	A	
rs7158238	NC_000014.9:g.92070761 C > T	A	G	G	
rs12588287	NC_000014.9:g.92070615 T > C	G	A	A	
rs7153615	NC_000014.9:g.92070595 C > T	A	G	G	
rs7153603	NC_000014.9:g.92070572 C > T	A	G	G	
rs7153696	NC_000014.9:g.92070452 G > A	T	C	C	
rs7153374	NC_000014.9:g.92070423 C > T	A	G	G	
rs7153193	NC_000014.9:g.92070350 C > A	T	G	G	
rs4904833	NC_000014.9:g.92069317 A > G	C	T	T	
rs7146985	NC_000014.9:g.92069172 G > A	T	C	C	
rs113572439	NC_000014.9:g.92069100 A > T	A	T	T	
rs2006047	NC_000014.9:g.92068664 T > C	G	G	G	
rs146668789	NC_000014.9:g.92068591 G > A	C	C	C	
rs8004149	NC_000014.9:g.92068571 C > T	A	G	G	
rs111735934	NC_000014.9:g.92068539 C > T	G	A	A	
rs181752420	NC_000014.9:g.92068435 G > A	C	C	C	
rs7142326	NC_000014.9:g.92068397 T > C	G	G	G	
rs74071847	NC_000014.9:g.92068329 G > A	C	C	C

**Table 2:** Age estimation of the Machado lineage in MJD families based on the genotyping of 7 STRs flanking the *ATXN3*_CAG repeat

Haplotype	No families	Mutation steps (n)	Age ± *δ* (years)	
			ε = 0.0105^1^	ε = 0.0122^2^
H1: **10–20-10–18–7–19–24**	47	0	3724 ± 804	3211 ± 693
H2: 10–20– 9–18–7 –19–24	7	1		
H3: 10–19–10–18–7 –19–24	1	1		
H4: 11–24– 9–18–7–19–24	6	1		
H5: 16–21–9–18–7–19–24	1	1		
H6: 16–23–9–18–7–19–24	1	3		
H7: 16–25–10–18–7–19–24	9	6		
H8: 17–25–10–18–7–19–24	5	7		
H9: 17–26–10–18–7–19–24	1	8		
H10: 17–25–10–19–7–19–24	1	8		
H11: 16–19– 9 –13–7–19–26	1	4		
H12: 10–20–10–18–7–15–24	2	1		

δ—Standard deviation

ε—Probability of change *per* generation

^1^Recombination rate (θ) based on the physical distance between the two most distant STRs

^2^Recombination rate (θ) based on the recombinant haplotypes observed in families

Age of the MJD lineage shared by this family was estimated, with both mutation (μ) and recombination (θ) rates included as generating variation, as detailed before (Martins et al. 2007). The estimated probability of mutation per generation and per STR (µ) was 4.79 × 10^–3^, taking into account that we analysed 4 di, 2 tri and 1 tetranucleotide STRs—the mutation rate for trinucleotides (6.13 × 10^−4^) was estimated as the median value between that for di (7.8 × 10^−4^) and tetranucleotide repeats (1.5–2.0 times lower than for dinucleotides) (Chakraborty et al. 1997; Gyapay et al. 1994). Recombination rate (θ) was based on (1) the physical distance between the 2 most distant markers (414 kilobases; θ = 0.0058 cM), with a conversion factor of 1 megabase = 1.41 cM; and (2) the observed meiotic events (2 recombinations in 268 meioses; θ = 0.0075 cM) (Martins et al. 2007). The probability of change per generation (*ε*) is given by *ε* = *1 − [(1 − θ)(1 − μ)]*, and the average of mutation and recombination events (*λ*) equals *εt*, where *t* is the number of generations (Martins et al. 2007).

## Results

### Clinical presentation

Patients F49-396 (the proband) and F49-398 were sibs born to non-consanguineous parents (Fig. 1), who presented with abnormal gait and speech in their second decade. The disease started in patient F49-396, at age 24, with gait ataxia, muscle cramps and spasticity, followed by dysarthria, by age 26 years. One year later, he could walk only with support, and became wheel-chair bound by age 29. He also complained of urinary urgency and mild dysphagia. On clinical examination (age 33 years), he had severe spasticity and hyperreflexia in upper and lower limbs, and bilateral Babinski sign. He also had mild muscle wasting in proximal upper limbs and moderate wasting in his lower limbs. His power was grade five in the upper and lower limbs. Also significant were mild upper-limbs ataxia and slow saccades. He could not perform heel-to-shin, due to the severe spasticity. Disease evolved until the age of death, at 38 years old.

In his sib, F49-398, onset was at age 20, with gait ataxia. One year later, he still walked independently, but had lost the ability to run, developed dysarthria and started having muscle cramps. Examined at age 24 years, this patient had hyperreflexia without spasticity in upper limbs, and severe spasticity and hyperreflexia in lower limbs and bilateral Babinski sign. His lower-limbs’ muscles were mildly wasted, but had a grade five power in upper and lower limbs. He showed moderate upper-limbs and mild lower-limbs ataxia. He had slow saccades, but apparently no limitation of eye movements. The time from the first symptoms to death was 7 years. Neither of the patients had bulging eyes, nor sensory or extrapyramidal involvement, including dystonic posturing.

### MJD lineage (SNPs) and flanking STR haplotypes

An expanded *ATXN3*_(CAG)_n_ allele was observed in each patient: F49-396 (29/74) and F49-398 (29/75) (Fig. 1). By segregation analysis, we were able to identify the allelic phase of the polymorphic markers with expanded alleles and construct haplotypes. We observed a “pure” Machado lineage associated with the MJD allele, covering a 15 kb region (Table 1, Fig. 1).

Previously, we studied SNP-based MJD haplotypes in more than 20 populations (Martins et al. 2007, 2012; Martins and Sequeiros 2018; Sharony et al. 2019); for this study, we analysed our current cohort of 393 MJD families, from 33 populations and identified the Machado lineage in 90 MJD families worldwide: 86 from Portugal, 2 from Spain and 2 from the USA (with no Portuguese ancestry). In addition, 3 Portuguese-Azorean families shared 29 of those 30 SNPs (differing only by allele C_rs12895357), but their flanking STR haplotype (H7) was common in other Machado families (which led us to hypothesize a recent recurrent back mutation G > C at rs12895357 for that sublineage). Therefore, a single origin should be shared by all 93 families and this Sudanese family, a total of 94 families for further analysis with the 7 flanking STRs.

We were, thus, able to reconstruct MJD-associated haplotypes in the Sudanese patients (H11:16-19-9-(CAG)_exp_-13-7-19-26), as well as in 79 other families of the Machado lineage (Table 2). The two Spanish families, although not reaching the threshold of 0.6 for the probability inferred (0.551 and 0.541) by the PHASE software (Stephens et al. 2001), shared the same 10-20-10-(CAG)_exp_-18–7-15–24 haplotype, the reason why we included them in this analysis. The 12 remaining families were excluded, as their disease-associated STR-haplotype could not be reliably inferred.

We observed a low gene diversity (0.204, SD = 0.139) in the Machado lineage, with only 12 STR-haplotypes being found among 82 families (Fig. 2). A founder haplotype (H1:10–20-10-(CAG)_exp_-18-7-19-24) was shared by 47 families, all of Portuguese origin. The two Spanish families showed a haplotype phylogenetically close to H1, probably resulted from a recombination in the founder haplotype. A recombination event (instead of stepwise mutations in STRs) is also the most parsimonious option to explain the origin of H4 and H5 from H1, since their downstream haplotype (4 STRs) is shared by all of them, with phylogenetically distant haplotypes observed only upstream.

Currently, in control populations, alleles 10_TAT223, 10_ATA194, 7_AAAC123, 19_GT190 and 24_AC190 are among the most frequent in all Europeans, Asians and Africans; it is noticeable, however, the very low frequency of allele 20_GT199 (5.7% in Europe (n = 405), 3% in Asia (n = 122), and 0% in Africa (n = 96)) and allele 18_AC21 (0.7% in Europe (n = 420); 3% in Asia (n = 119), and 0% in Africa (n = 97)).

### Age (re)estimation of the Machado lineage

We have currently analysed a total of 393 MJD families (from 33 populations; including unpublished data), and could draw a broad picture of MJD lineages (and sublineages) worldwide. This Sudanese family with MJD shares the ancestral (“pure”) Machado lineage, present in 24% of all families studied. Thus, we estimated the time ensued from their likely common ancestor. As the most ancient STR haplotypic background, we assumed H1 to be the founder core haplotype of the network (Fig. 2). From there, we estimated the number of stepwise STR mutations and recombinations that would be implied to originate the remaining STR haplotypes (Table 2).

To capture an accurate picture on the effect of recombination on age estimation, we calculated the recombination fraction for these STRs, based on (1) physical distance between the two farthest STRs typed (θ = 0.0058 cM); and (2) meiotic events (2 recombinations observed among 268 meioses; θ = 0.0075 cM). Thus, assuming a generation time of 25 years, and based on the most accurate recombination estimates (from family data), we estimated the Machado lineage to be 3,211 ± 693 years.

## Discussion

MJD has been reported from Africa and in a few families of African descent in the USA. To the best of our knowledge, these are the first MJD patients described in Sudan. Previous studies, including only three core intragenic SNPs, showed patients with African descent to have ACA, AGA or GCC (rs1048755–rs12895357–rs7158733) haplotypes, but not the GGC core haplotype that defines the Machado lineage (reviewed in Ogun et al. 2015; Subramony et al. 2002). This family shows the same exact (“pure”) Machado lineage, as the MJD families originating from Portugal (primarily central mainland and the Azorean island of São Miguel).

At the phenotypical level, however, onset (both in the proband and his examined brother) was at a relatively early age (24 and 20 years old), and time of disease evolution was short (14 and 7 years, respectively). In the Portuguese-Azorean islands of São Miguel and Flores (homelands of the Machado and Joseph families), as well as in Portuguese migrants in the USA and Canada, there is a well-established difference in AO and disease duration: onset occurs later and disease course is more protracted in the Machado family and others from São Miguel and in the east coast of the USA; mean AO ± SD was 43.2 ± 13.5 in patients from São Miguel (*versus* 35.3 ± 15.2 in Flores) (Sequeiros 1989). Accordingly, MJD subphenotypes 3 and 1 were predominantly observed in Machado and Joseph original families, respectively (Coutinho 1992; Nakano et al. 1972; Rosenberg et al. 1976; Sequeiros 1989); our current knowledge on the distinct haplotype backgrounds of these two families sheds light on what could have been one of the first phenotype-genotype studies in MJD. Still nowadays, such correlations are scarce and difficult to perform since (1) a precise clinical examination is required with patients observed for more than seven years, once types 1 and 3 are considered to be defined; and (2) sometimes, more than one subtype is found within the same family, mainly across generations. The Sudanese brothers analysed in this study showed earlier onset and a more severe and rapid disease progression, relatively to the Portuguese kindreds of the Machado lineage. Interestingly, in Huntington’s disease (HD), a high frequency of juvenile-onset cases reported in the Middle-East has been associated with a unique SNP-based HD haplotype of African origin (Squitieri et al. 2020). Contrarily to HD, however, the earlier onset in these Sudanese MJD patients does not seem to be explained by large CAG repeat expansions; other *cis* or *trans*-acting modifiers present in their population background, and/or environmental or stochastic factors may account for it.

The MJD Machado lineage had only been observed in Portugal and in a few countries with well-documented connections to Portugal (as nearby Spain, and migrants’ descendants in Brazil and North-America), even if only three or six SNPs were typed in those studies (Gaspar et al. 2001; Martins et al. 2007). Now, we genotyped 30 intragenic SNPs and 7 STRs flanking the expanded (CAG)_n_, in this Sudanese family and in the other 93 MJD families sharing its Machado ancestral origin. The analysis of phylogenetic relationships among STR haplotypes, supported a Portuguese ancestry for the Machado lineage. On the other hand, a *recent* migration of expanded alleles from the population-of-birth to Sudan does not seem plausible since there was no reported Portuguese ancestry in the family, and the most parsimonious phylogenetic relationship between H11 (present in this Sudanese family) and H5 (found in Portuguese patients) implies one recombination plus 2 STR mutation steps (Table 2). Therefore, dispersal routes responsible for the presence of MJD in Sudan are difficult to trace, since no intermediate STR haplotypes were found. When comparing STR haplotypes among all families with the Machado lineage, we see 4 STRs downstream to be highly conserved, with only one single-step mutation at AC_21 (H10) and two recombination events (H11 and H12). This reinforces a single recent mutational origin for all, rather than a predisposing haplotype; accordingly, two alleles of the ancestral haplotype H1 are extremely rare in European and Asian control populations or even absent among Africans. Thus, assuming H1 as the ancestral STR haplotype, 5 branches diverge from it in the most parsimonious network. Origin of H4 and H5 by recombination from H1 is also the best explanation, as the alternative of their evolution by the many stepwise mutations required (on the 3 STRs upstream) seems more unlikely.

The fact that most Portuguese families with this lineage share the ancestral haplotype H1, strongly suggests that a major founder effect contributed to the high frequency of MJD in Portugal, as previously suggested (Martins et al. 2007). The main founder Machado haplotype seems to coincide with the ancestral background where a de novo expansion might have occurred.

In spite of our extensive search in many other populations, the full picture of the Machado mutational origin may yet be incomplete, as MJD could still being underdiagnosed in some populations from Africa and elsewhere. If that were the case, the age of 3211 ± 693 years, as now anticipated for the ancestral origin of this lineage, would be an underestimate. In any case, as previously postulated (Martins et al. 2007), the mutational origin of the Machado lineage must be much more recent than the worldwide-spread Joseph lineage (5,774 ± 1116y (Martins et al. 2007), 16,335 ± 1966y (Li et al. 2018)), or the Joseph-like sublineages (11,837 ± 1871y, 9272 ± 1352y, 9254 ± 1411y, all estimations in the Chinese population (Li et al. 2018); and 7191 ± 1252y for the reported Joseph-Groote sublineage found in several Asian families (Martins et al. 2012)). The use of the information provided by the genotyping of Ancestry Informative Markers in this genomic region could provide useful clues to clarify the origin of these lineages in terms of their continental birthplaces.

## Data Availability

All relevant data analysed during this study are included in this published article. Additional datasets generated during the current study are available from the corresponding author on reasonable request.
